# Coping strategies of women with postpartum depression symptoms in rural Ethiopia: a cross-sectional community study

**DOI:** 10.1186/s12888-018-1624-z

**Published:** 2018-02-08

**Authors:** Telake Azale, Abebaw Fekadu, Girmay Medhin, Charlotte Hanlon

**Affiliations:** 1 University of Gondar, College of Medicine and Health Sciences, Department of Health Education and Behavioral Sciences, Gondar, Ethiopia; 2 Addis Ababa University, Department of Psychiatry, School of Medicine, College of Health Sciences, 9086 Addis Ababa, PO Ethiopia; 3 King’s College London, Institute of Psychiatry, Psychology and Neuroscience, Department of Psychological Medicine, Centre for Affective Disorders, London, UK; 4 Addis Ababa University, Aklilu Lemma Institute of Pathobiology, Addis Ababa, Ethiopia; 5 King’s College London, Institute of Psychiatry, Psychology and Neuroscience, Centre for Global Mental Health, London, UK

## Abstract

**Background:**

Most women with postpartum depression (PPD) in low- and middle-income countries remain undiagnosed and untreated, despite evidence for adverse effects on the woman and her child. The aim of this study was to identify the coping strategies used by women with PPD symptoms in rural Ethiopia to inform the development of socio-culturally appropriate interventions.

**Methods:**

A population-based, cross-sectional study was conducted in a predominantly rural district in southern Ethiopia. All women with live infants between one and 12 months post-partum (*n* = 3147) were screened for depression symptoms using the validated Patient Health Questionnaire, 9 item version (PHQ-9). Those scoring five or more, ‘high PPD symptoms’, (*n* = 385) were included in this study. The Brief Coping with Problems Experienced (COPE-28) scale was used to assess coping strategies. Construct validity of the brief COPE was evaluated using confirmatory factor analysis.

**Results:**

Confirmatory factor analysis of the brief COPE scale supported the previously hypothesized three dimensions of coping (problem-focused, emotion-focused, and dysfunctional). Emotion-focused coping was the most commonly employed coping strategy by women with PPD symptoms. Urban residence was associated positively with all three dimensions of coping. Women who had attended formal education and who attributed their symptoms to a physical cause were more likely to use both problem-focused and emotion-focused coping strategies. Women with better subjective wealth and those who perceived that their husband drank too much alcohol were more likely to use emotion-focused coping. Dysfunctional coping strategies were reported by women who had a poor relationship with their husbands.

**Conclusions:**

As in high-income countries, women with PPD symptoms were most likely to use emotion-focused and dysfunctional coping strategies. Poverty and the low level of awareness of depression as an illness may additionally impede problem-solving attempts to cope. Prospective studies are needed to understand the prognostic significance of coping styles in this setting and to inform psychosocial intervention development.

**Electronic supplementary material:**

The online version of this article (10.1186/s12888-018-1624-z) contains supplementary material, which is available to authorized users.

## Background

Postpartum depression (PPD) is a major public health concern in low and middle income countries (LMICs) [1]. PPD is the most common mental health problem in the perinatal period, with a weighted mean prevalence estimate of 19.8% (95% confidence interval 19.5 to 20.0%) in community studies from LMICs [2]. Risk factors for PPD in LMICs are mostly social stressors, including poverty, poor social support, intimate partner violence, history of pregnancy loss and unintended pregnancy, all of which tend to be common in LMICs [2]. Evidence for a negative impact of PPD in LMICs is accumulating [3]. Many studies have shown that the children of depressed mothers are more likely to be undernourished [4], although evidence from sub-Saharan Africa is conflicting [5–7]. Increased risk of infant diarrheal episodes has been found in Ethiopia [8] and Pakistan [9], and increased infant mortality associated with maternal depression has been shown in Ethiopia [10] and Ghana [11]. Adverse effects of maternal depression on child development have been found in India [12] and Bangladesh [13], with conflicting reports from Ethiopia [5, 14]. Despite these adverse effects, PPD in women in LMICs mostly remains undetected and untreated.

There is a global initiative to integrate mental health services into maternal health care [15]. The World Health Organization has identified evidence-based packages of care for depression and other priority mental disorders which can be delivered by general health workers [16]. Given the challenges associated with safety and acceptability of psychotropic medicines in the perinatal period, there is a need for contextually appropriate psychosocial interventions [17, 18]. There is burgeoning evidence for effective psychosocial interventions for PPD that can be provided by non-mental health professionals in middle-income countries [19–21], but little from rural, low income country settings. Adaptation and successful implementation of a psychosocial intervention requires an understanding of women’s existing coping mechanisms within that context.

Coping is described as the cognitive and behavioral processes that a person uses to deal with stressful circumstances that are judged to be demanding, challenging, threatening and/or have a potential for harm or loss [22]. Coping styles have been categorized into three dimensions: problem-focused, emotion-focused and dysfunctional or avoidance coping [22, 23]. Problem-focused coping involves dealing with the source of stress, while emotion focused-coping is when the person attempts to handle thoughts and feelings associated with the stressor [22]. Avoidance or dysfunctional coping refers to strategies which avoid dealing with either the problem or the associated emotions [23]. The choice of coping strategies also depends on the nature of the stressor, the person’s perception of the seriousness of the experience and his/her evaluation of the resources available for coping and the likely effectiveness of a given strategy [22]. The negative cognitive biases and self-concept seen in individuals with depression may weaken their use of adaptive coping strategies and thereby influence outcome [24]. In support of this, associations have been found between coping strategies and the onset, course and outcome of depression in high-income countries [25]. People with depression are more likely to use avoidance coping strategies [25, 26]. Problem solving and emotion-focused coping are associated with less severe symptoms, while venting emotions (a dysfunctional strategy) has been associated with more impairment in functioning [27].

Coping strategies vary across cultures [28, 29]. There have been few studies examining the association between perinatal depression and coping strategies. Perinatal depression has been associated with emotion-focused coping in Greece [30] and with avoidance or dysfunctional coping in Belgium [31], Spain [32] and Greece [33]. In a study from a middle-income country, Brazil, an association was found between PPD and avoidance coping [34]. A study of coping among Ethiopians displaced from Eritrea found that both women and those who experienced traumatic events used emotion-focused coping more frequently, although female sex and better social support were associated with problem-focused coping [35].

The lack of evidence on coping strategies employed by women with PPD in a rural, low-income country setting, like Ethiopia, is a barrier to scaling up appropriate care. In Ethiopia and four other LMICs, the PRogramme for Improving Mental health carE (PRIME) is working to integrate maternal mental health services into primary health care. The aim of the current study was to assess the specific coping strategies used by women with symptoms of PPD in a rural Ethiopian setting with a view to informing development of appropriate interventions.

## Methods

### Study design

A population-based cross-sectional survey.

### Study area

The study was conducted in Sodo district, Gurage Zone, Southern Nations, Nationalities and Peoples’ Region (SNNPR) of Ethiopia. Sodo is located about 100 km south of the capital city, Addis Ababa. In the most recent census, the population was estimated to be 161,097 (79,896 male and 81,201 female) [36]. About 85% of the population lives in a rural setting. Amharic is the official language of the region but is the second language for the majority of inhabitants. Within Sodo district, there are eight primary care health centers, each linked to health posts which are staffed by community-based health extension workers.

The nearest psychiatric out-patient service is located in Butajira town, 30 to 50 km away. At the time of the study there was no specialist mental health professional located within the district. As part of the PRogramme for Improving Mental health carE (PRIME) [37], health centre staff, including midwives, were given brief in-service training to deliver mental health care in the primary care setting, supported by periodic supervision by a psychiatric nurse. At the time of data collection, the new service was not yet operational.

### Sampling

A population census was conducted by the PRIME project. The census was used to identify women with infant children (< 1 year of age). To augment this strategy, a list of immunized infants was obtained from a registry maintained by the health extension workers. As a further strategy, data collectors, who had been recruited from the local community, went house to house to identify any additional eligible women. A total of 3147 eligible women were identified. As part of an interview assessment (described below), all women were screened for PPD symptoms using the Patient Health Questionnaire (PHQ-9). Those scoring above the validated cut-off indicating depression (‘high PPD symptoms’) were included in the study. The eligibility criteria were as follows:

women between one and 12 months postpartum, able to converse in Amharic (the official language of the region) and not acutely unwell.

### Data collection

Data were collected using a structured questionnaire that included socio-demographic, obstetric, psychosocial and behavioral variables. Data collectors (*n* = 36) were trained for nine days and interviewed women in their homes. Four supervisors were also trained and assisted by the investigators. The data collectors were recruited from the district and the sub-districts or, in cases where eligible persons were not available at the specific sub-district, from the neighboring sub-districts. A pretest was conducted in three sub-districts of another district near the study area.

Each household containing an eligible woman was visited by a data collector who then explained the purpose of the research and gave the woman an information sheet or read the information aloud for those who were unable to read. Women who consented to participate were interviewed at a time and place that was convenient for them. Following screening with PHQ-9, those women scoring 5 or more were administered additional questionnaires. Data were collected between April and June 2014.

### Measures

#### Coping strategies

In this study we used the Brief COPE Scale [38] to measure coping strategies. The brief COPE is a shortened version of the COPE scale, and has been used widely, including in LMICs.

In validation studies, the Brief COPE Scale was found to have reasonable reliability and validity [39–46]. Although the brief COPE has been used in more than 400 studies, it has rarely been used to assess coping in people with depression. For example, a systematic review of 80 studies that used the Brief COPE revealed that none of the studies was on people with depression [47].

The Brief COPE was originally developed to measure coping with any stressor [38]. The scale has 28 items that assess the degree to which a respondent utilizes a specific coping strategy. The 28 items have been categorized into 14 coping strategies named as: active coping, planning, positive reframing, acceptance, humor, religion, using emotional support, using instrumental support, self-distraction, denial, venting, substance use, behavioral disengagement and self-blame [38] and three higher order categories known as problem-focused, emotion-focused [22, 23], and avoidance or dysfunctional coping [23]. Alternatively, the brief COPE items have been categorized into “adaptive” and “maladaptive” coping. The development of these sub-scales was conceptually driven, and the theoretical grouping of the items to their sub-scales was largely established using principal components analysis [23]. Respondents rate items on a 4-point Likert scale, ranging from 1 “*I haven’t been doing this at all*” to 4 “*I’ve been doing this a lot*”. As most of the participants were not literate, the items were rephrased into questions so that the questionnaire became interviewer administered. For example, *“have you been turning to work or other activities to take your mind off things?”* for I have been turning to work or other activities to take my mind of things.

PPD was measured using the Patient Health Questionnaire (PHQ-9) [48]. This instrument was developed to measure depression in the Primary Health Care (PHC) setting. The PHQ-9 has been culturally validated for use in several African country settings [49–53], including postpartum women in a rural setting in Ghana [54], and in the primary health care context in rural Ethiopia [55]. In Ethiopia, a score of five or more was found to have a sensitivity of 83% and specificity of 75% for the detection of major depressive disorder.

Explanatory models for PPD symptoms were measured with the Short Explanatory Model Interview (SEMI) [56]. The woman’s perception of causes, severity, treatment need and options for symptoms of PPD were assessed by asking open-ended questions and coding the responses using pre-specified categories which had been adapted for the socio-cultural context in Ethiopia. Perceived severity of the condition was coded using the following categories: unable to say, not at all serious, mildly serious, moderately serious or very serious.

Disability was measured using the World Health Organization Disability Assessment Schedule (WHODAS) [57]. Disability was measured in six domains of functional impairment, including understanding and communicating, getting around, self-care, getting along with people, life activities, and participation in society. Scores were from 1 (no difficulty) to 5 (extreme or cannot do). The instrument has been used previously in perinatal women in a rural Ethiopian setting and found to have convergent validity with depressive symptoms [58].

Social support was measured using the Oslo Social Support Scale (OSSS-3) [59], which measures strength of social support. Scores from 3 to 8 indicate poor support, scores from 9 to 11 intermediate support, and a score between 12 and 14 is considered indicative of strong social support. The OSSS-3 has been used in community studies in Ethiopia previously [60].

The following potential confounders were measured using questions which have been used previously in postnatal women in the neighboring district: educational level, perceived relative wealth and hunger due to lack of resources in the past month (socio-economic status), quality of marital relationship, perception that the husband drank too much alcohol or chewed too much khat, and rural or urban residence.

### Data management and analysis

Data were double entered using EpiData version 3.1 and then exported to SPSS version 20, cleaned, coded and analyzed. Descriptive analyses were carried out, including frequencies, mean and standard deviation and correlations among the items and sub-scales of the Brief COPE. Frequencies of the 28 items of the Brief COPE scale were calculated into the four Likert categories. The internal consistencies of the higher order scales were calculated with Cronbach’s alpha. Confirmatory factor analysis was carried out to confirm the recommended three dimensions of coping namely, problem-focused, emotion-focused, and dysfunctional or avoidant coping [22, 61] using STATA 12 and AMOS-23 software. The most frequently used model fit indices include chi-square, root mean square error of approximation (RMSREA), comparative fit index (CFI), and Tucker-Lewis Index (TLI). Although there is no agreement as to the acceptable values of the indices, values closer to 1 are considered good fit if TFI and CLI. A value closer to zero is better for RMSEA. The chi-square test is frequently used as an index of model fit. A smaller chi-square value which is insignificant at *p* = 0.05 is considered to be a good fit. However, the value of chi-square is affected by several factors, such as sample size, the number of variables, and distribution of variables. Hence it is said to not be a good index practically. This is the reason for the use of alternative indices [62].

The 28 items of the Brief COPE scale were summed into 14 subscales after confirmatory factor analyses. Simple and multiple linear regression analyses were then carried out to examine factors associated with each of the three dimensions of coping. A priori confounders were included in the model, as described above. Other items of exploratory interest, e.g. from the SEMI, were included in the multivariable model if the univariate association with coping strategies had *p* < 0.2).

### Ethical considerations

Ethical approval was obtained from the Institutional Review Board (IRB) of the College of Health Sciences, Addis Ababa University. Permission letters were received from the district health and administrative offices. Eligible women were given information sheets and those who agreed to participate signed or gave their finger prints before they were interviewed. Women who expressed suicidal ideation were put in contact with mental health care.

## Results

There was a 100% response rate in the women screened for PPD symptoms. A total of 385 women with a PHQ-9 score of 5 or more were included in the analyses. The percentage of women with high PPD symptoms was, therefore, 12.2% (385/3147). One woman was excluded due to probable psychotic symptoms.

### Socio-demographic and psychosocial characteristics

The mean PHQ score of the participants was 7.8 (Standard Deviation (SD): 3.03). The mean age of the respondents was 28.8 years with 5.23) standard deviation. More than half (*n* = 232; 60.3%) of the respondents were between 25 and 34 years of age. Almost all, (*n* = 374; 97.1%) were married. Three hundred and sixty-three women (94.3%) were rural residents. More than two-thirds, (*n* = 271; 70.4%) were unable to read and write. The vast majority, (*n* = 365; 94.8%) of the respondents were followers of Orthodox Christianity and 353 (91.7%) were Gurage by ethnicity. See Table 1.

**Table 1:** Socio-demographic and psychosocial characteristics of women with symptoms of postpartum depression, Sodo district, 2014 (*n* = 385)

Socio-demographic characteristic	Frequency (%)
Age (in years)	
Less than 20	28 (7.3)
20–29	174 (45.2)
30–39	175 (45.5)
40 or above	8 (2.1)
Marital status	
Married	374 (97.1)
Single/widowed/divorced	11 (2.9)
Residence	
Rural	363 (94.3)
Urban	22 (5.7)
Education	
No formal education	308 (80.0)
Formal education	77 (20.0)
Religion	
Orthodox	365 (94.8)
Protestant	12 (3.1)
Muslim	6 (1.6)
Other	2 (0.6)
Ethnicity	
Gurage	353 (91.7)
Oromo	26 (6.8)
Amhara	5 (1.3)
Other	1 (0.3)
Psychosocial characteristics	
Hunger in the past one month	
No	259 (67.3)
Yes	126 (32.7)
Relative wealth	
Less	171 (44.4)
Same	198 (51.4)
Better	16 (4.2)
Social support	
Poor	104 (27.0)
Intermediate	187 (48.6)
Strong	94 (24.4)
Husband drinks too much	
No	290 (75.3)
Yes	95 (24.3)
Quality of marital relationship	
Bad	80 (20.8)
Good	305 (79.2)
Perceived cause of postpartum depression symptoms	
Psychosocial	231 (60.0)
Physical	89 (23.1)
Supernatural	16 (4.2)
Don’t know	49 (12.8)
Perceived severity of PPD symptoms	
Mild	81 (21.0)
Moderate	151 (39.2)
Severe	153 (39.8)
WHODAS score (Disability)	
None	28 (7.3)
Mild	212 (55.1)
Moderate	126 (32.7)
Severe	18 (4.7)
Extreme or cannot do	1 (0.3)

### Coping strategies

Religious coping was the most frequently used strategy. The two items: “*I have been praying or meditating”* and “*I have been trying to find comfort in my religion or spiritual beliefs”* were reported to be used ‘a lot’ by 49.9% (*n* = 192) and 36.4% (*n* = 140) respondents, respectively. The least frequently used coping strategies were substance use (*n* = 8; 2.1%) and humor 4.2% (*n* = 16). See Table 2.

**Table 2:** Frequency of use of coping strategies by women with symptoms of PPD (n = 385)

Coping strategies	Mean(SD)	Not at all or a little bit	A medium amount	A lot
Emotion-focused coping strategies				
I’ve been praying or meditating.	2.24 (0.83)	96 (24.9)	97 (25.2)	192 (49.9)
I’ve been trying to find comfort in my religion or spiritual beliefs.	1.95 (0.87)	156 (40.5)	89 (23.1)	140 (36.4)
I’ve been getting comfort and understanding from someone.	1.84 (0.84)	171 (44.4)	102 (26.5)	112 (29.1)
I’ve been accepting the reality of the fact that it has happened.	1.48 (0.76)	261 (67.8)	61 (15.8)	63 (16.4)
I’ve been looking for something good in what is happening.	1.46 (0.75)	269 (69.9)	54 (14)	62 (16.1)
I’ve been trying to see it in a different light, to make it seem more positive.	1.44 (0.74)	273 (70.9)	53 (13.8)	59 (15.3)
I’ve been learning to live with it.	1.37 (0.67)	284 (73.8)	59 (15.3)	42 (10.9)
I’ve been making jokes about it.	1.10 (0.42)	361(93.8)	8(2.1)	16(4.2)
I’ve been making fun of the situation.	1.12 (0.42)	359(93.2)	10(2.6)	16(4.2)
Problem-focused coping strategies				
I’ve been taking action to try to make the situation better.	1.79 (0.82)	178 (46.2)	109 (28.3)	98 (25.5)
I’ve been trying to get advice or help from other people about what to do.	1.65 (0.82)	219 (56.9)	78 (20.3)	88 (22.9)
I’ve been concentrating my efforts on doing something about the situation I’m in.	1.63 (0.80)	222 (57.7)	82 (21.3)	81 (21)
I’ve been thinking hard about what steps to take.	1.67 (0.79)	206 (53.5)	100 (26)	79 (20.5)
I’ve been trying to come up with a strategy about what to do.	1.62 (0.79)	221 (57.4)	88 (22.9)	76 (19.7)
I’ve been getting help and advice from other people.	1.41 (0.71)	276 (71.7)	58 (15.1)	51 (13.2)
I’ve been getting emotional support from others.	1.48 (0.78)	269 (69.9)	47 (12.2)	69 (17.9)
Avoidance/ dysfunctional coping strategies				
I’ve been turning to work or other activities to take my mind off things.	1.68 (0.87)	288 (59.2)	50 (13)	107 (27.8)
I’ve been doing something to think about it less, such as going to movies, watching TV, reading, daydreaming, sleeping, or shopping.	1.73 (0.86)	207 (53.5)	73 (19)	105 (27.3)
I’ve been saying things to let my unpleasant feelings escape.	1.52 (0.77)	250 (64.9)	68 (17.7)	67 (17.4)
I’ve been blaming myself for things that happened.	1.54 (0.77)	242 (62.9)	76 (19.7)	67 (17.4)
I’ve been criticizing myself.	1.51 (0.76)	253 (65.7)	67 (17.4)	65 (16.9)
I’ve been saying to myself “this isn’t real”.	1.38 (0.70)	287 (74.5)	47 (12.2)	51 (13.2)
I’ve been expressing my negative feelings.	1.37 (0.69	291 (75.6)	45 (11.7)	49 (12.7)
I’ve been refusing to believe that it has happened.	1.34 (0.69)	300 (77.9)	37 (9.6)	48 (12.5)
I’ve been giving up trying to deal with it.	1.26 (0.60)	316(82.1)	37(9.6)	32(8.3)
I’ve been giving up the attempt to cope.	1.21 (0.55)	329(85.5)	30(7.8)	26(6.8)
I’ve been using alcohol or other drugs to make myself feel better.	1.08 (0.37)	366(95.1)	6(1.6)	13(3.4)
I’ve been using alcohol or other drugs to help me get through it.	1.06 (0.31)	369(95.8)	8(2.1)	8(2.1)

Scores on the problem-focused coping sub-scale ranged from six to 24, with a mean of 14.6 (SD 3.95). The emotion-focused coping score ranged from 10 to 40, with a mean of 21.4 (SD 5.41). Dysfunctional coping scores ranged from 12 to 48 with a mean of 22.6 (SD 6.07).

### Construct validity of the brief COPE

The internal consistency of each sub-scale was good, with Cronbach’s alpha between 0.72 and 0.74. In the confirmatory factor analysis, the coefficients for items loading onto the three dimensions of coping ranged from 0.38 for substance use (*I have been using alcohol or other drugs to help me get through it*) to 0.82 for self-blame (*I have been blaming myself for things that happened*) and religion (*I have been trying to find comfort in my religion or spiritual beliefs*). The inter-item correlations were all significant except for the religion and humor items. The three higher order categories (problem-focused, emotion-focused and dysfunctional coping) were highly correlated. The CFA model fit indices were as follows: CMIN/DF 3.58, root mean squared error of approximation (RMSEA) 0.02, the comparative fit index 0.72, and Tucker Lewis Index 0.65. Removing the items with low coefficients did not improve the model fit. Therefore, all items were retained for further analysis. (See Additional file 1).

### Factors associated with problem-focused coping

In the univariate analysis, urban residence, formal education, better relative wealth and attribution of symptoms to a physical cause were all associated significantly with higher scores on the problem-focused coping sub-scale. Not knowing the cause of the illness was associated with significantly lower problem-focused coping scores.

All except relative wealth remained significantly associated in the multivariable model. See Table 3.

**Table 3:** Simple and multiple linear regression analysis of factors associated with problem focused coping among women with symptoms of postpartum depression, (*n* = 385)

	Mean (SD)	Crude β (95% CI)	Adjusted β (95% CI)
Age, mean (SD), 28.8(5.2)		−0.00 (− 0.08, 0.07)	0.02 (− 0.04, 0 .10)
Residence			
Rural	14.4 (0.2)	Ref	Ref
Urban	17.3 (0.7)	**2.96 (1.28, 4.65)**	**2.39 (0.67, 4.11)**
Educational Status			
No formal education	14.2 (0.2)	Ref	Ref
Formal education	15.8 (0.4)	**1.53 (0.55, 2.51)**	**1.50 (0.44, 2.56)**
Hunger in the preceding month			
No	14.7 (0.2)	Ref	Ref
Yes	14.1 (0.3)	−0.63 (− 1.48, 0.20)	−0.47 (− 1.46, 0.50)
Subjective relative wealth			
Less	14.0 (4.2)	Ref	Ref
Same	14.8 (3.5)	**0.84 (0.04, 1.65)**	0.80 (−0.14, 1.75)
Better	16.5 (4.5)	**2.53 (0.52, 4.55)**	1.62 (−0.36, 3.61)
Social support			
Poor	14.8 (3.9)	Ref	Ref
Intermediate	14.0 (3.6)	−0.76 (−1.71, 0.18)	− 0.91 (− 1.86, 0.04)
Strong	15.2 (4.5)	0.32 (−0.77, 1.42)	− 0.18 (− 1.33, 0 .95)
Husband drinks too much alcohol			
No	14.3 (0.2)	Ref	Ref
Yes	15.1 (0.4)	0.80 (−0.11, 1.71)	0.90 (0.00, 1.81)
Marital relationship			
Bad	14.9 (0.4)	Ref	Ref
Good	14.4 (0.2)	−0.45 (−1.43, 0.52)	− 0.45 (− 1.44, 0.53)
PHQ score		0.11 (− 0.02, 0.24)	0.12 (− 0.00, 0.25)
Perceived cause			
Psychosocial	14.5 (3.8)	Ref	Ref
Physical	15.6 (4.3)	**1.13 (0.18, 2.08)**	**1.12 (0.17, 2.07)**
Supernatural	13.8 (3.6)	−0.71 (− 2.69, 1.26)	−0.95 (− 2.94, 1.04)
Unable to say	12.9 (3.4)	**−1.54 (− 2.75, − 0.34)**	**−1.41 (− 2.62, − 0.21)**
Perceived severity			
Mild	14.8 (3.7)	Ref	Ref
Moderate	14.0 (3.8)	−0.75 (−1.82, − 0.31)	−0.61 (− 1.69, 0.46)
Severe	14.9 (4.2)	0.08 (−0.98, 1.14)	0.36 (− 0.74, 1.47)
WHODAS Score		−0.05 (− 0.16, 0.04)	−0.09 (− 0.20, 0.01)

bold type signifies statistical significance at *p* < 0.05

### Factors associated with emotion-focused coping

Urban residence, formal education, better wealth, perception that her husband drank too much alcohol, and perceived physical cause for the symptoms were associated positively with emotion-focused coping in both the simple and multivariable linear regression analyses. Not knowing the cause of the illness and WHODAS score were negatively associated with emotion-focused coping. See Table 4.

**Table 4:** Simple and multiple linear regression analysis of factors associated with emotion focused coping among women with symptoms of postpartum depression, (n = 385)

Characteristics	Mean(SD)	Crude β(95% CI)	Adjusted β(95% CI)
Age		0.00 (− 0.09, 0.10)	0.04 (− 0.05, 0.15)
Residence			
Rural	21.2 (0.2)	ref	ref
Urban	24.4 (1.1)	**3.18 (0.86, 5.50)**	**2.60 (0.28, 4.93)**
Educational Status			
No formal education	21.0 (0.3)	ref	ref
Formal education	22.9 (0.6)	**1.91 (0.56, 3.25)**	**2.02 (0.59, 3.46)**
Hunger			
No	21.6 (0.3)	ref	ref
Yes	20.8 (0.4)	−0.75 (−1.91, 0.40)	−0.34 (−1.69,1.00)
Relative wealth			
Less	20.8 (5.4)	Ref	ref
Same	21.5 (5.2)	0.72 (−0.37, 1.82)	0.74 (−0.54, 2.03)
Better	25.3 (6.5)	**4.53 (1.77, 7.28)**	**3.22 (0.53, 5.92)**
Social support			
Poor	21.4 (4.4)	Ref	ref
Intermediate	20.8 (4.9)	−0.56 (−1.85, 0.73)	−0.56 (−1.85, 0.72)
Strong	22.4 (6.9)	1.04 (−0.46, 2.55)	0.41 (−1.13, 1.95)
Husband drinks too much alcohol			
No	20.9 (0.3	ref	ref
Yes	22.8 (0.6)	**1.89 (0.64, 3.14)**	**(0.69, 3.14)**
Marital relationship			
Bad	21.8 (0.6)	ref	ref
Good	21.3 (0.3)	−0.59 (−1.93, 0.74)	− 0.52 (− 1.86, 0.81)
PHQscore (Mean:7.83, SD: 3.03)		0.11 (− 0.06, 0.29)	0.15 (− 0.02, 0.33)
Perceived cause			
Psychosocial	21.2 (4.9)	ref	ref
Physical	23.4 (6.2)	**2.21 (0.91, 3.50)**	**2.35 (1.06, 3.63)**
Supernatural	21.0 (6.2)	−0.18 (−2.86, 2.50	−0.61 (−3.31, 2.08)
Unable to say	18.9(4.6)	**−2.20 (−3.83, − 0.56)**	**−1.96 (−3.59, − 0.33)**
Perceived severity			
Mild	21.3 (4.9)	ref	ref
Moderate	21.1 (5.6)	−0.15 (−1.62, 1.31)	0.09 (−1.37, 1.55)
Severe	21.7 (5.4)	0.42 (−1.04,1.88)	0.65 (−0.85,2.15)
WHODAS Score		−0.13 (− 0.27, 0.00)	**−0.16 (− 0.30, − 0.02)**

bold type signifies statistical significance at *p* < 0.05

### Factors associated with avoidance/dysfunctional coping

Urban residence, better wealth, perception that her husband drank too much alcohol and bad marital relationship were positively associated with dysfunctional coping. Not knowing the cause of illness, and WHODAS score were negatively associated with dysfunctional coping in the simple linear regression. Relative wealth and perception that the husband drank too much alcohol were no longer significant in the multivariate analysis. See Table 5.

**Table 5:** Simple and multiple linear regression analysis of factors associated with dysfunctional coping among women with symptoms of postpartum depression, (*n* = 385)

Characteristics	Mean (SD)	Crude β (95% CI)	Adjusted β (95% CI)
Age		0.02 (− 0.09, 0.13)	0.07 (− 0.04, 0.19)
Residence			
Rural	22.4 (0.3)	Ref	Ref
Urban	26.0 (1.3)	**3.63 (1.03, 6.23)**	**4.13 (1.50, 6.76)**
Educational Status			
No formal education	22.5 (0.3)	Ref	Ref
Formal education	22.8 (0.7)	0.23 (−1.28, 1.75)	0.60 (− 1.02, 2.22)
Hunger			
No	22.9 (0.4)	Ref	Ref
Yes	21.9 (0.5)	−0.94 (−2.23, 0.35)	−0.92 (−2.45,0.60)
Relative wealth			
Less	22.2 (5.9)	Ref	Ref
Same	22.6 (5.8)	0.38 (−0.85,1.62)	0.28 (−1.17, 1.73)
Better	25.6 (9.5)	**3.34 (0.23, 6.45)**	1.72 (−1.32, 4.77)
Social support			
Poor	22.1 (5.5)	Ref	Ref
intermediate	22.4 (5.3)	0.30 (−1.15, 1.76)	−0.15 (− 1.61, 1.30)
Strong	23.4 (7.7)	1.25 (−0.44, 2.95)	0.11 (−1.63, 1.30)
Husband drinks too much alcohol			
No	22.2 (0.3)	Ref	Ref
Yes	23.8 (0.7)	**1.65 (0.24, 3.05)**	1.23 (−0.14, 2.62)
Marital relationship			
Bad	24.6 (0.6)	**2.54 (1.06, 4.02)**	**2.94 (1.44, 4.43**)
Good	22.0 (0.3)	**Ref**	**Ref**
PHQ score		0.09 (−0.10, 0.30)	0.11 (− 0.09, 0.31)
Perceived cause			
Psychosocial	22.6 (5.7)	Ref	Ref
Physical	23.7 (7.3)	1.03 (−0.44, 2.51)	1.39 (−0.05,2.84)
Supernatural	21.7 (5.0)	−0.92 (−3.99, 2.13)	− 0.56 (−3.61, 2.49)
Unable to say	20.6 (5.0)	**−2.02 (−3.88, − 0.15**)	**−1.97 (− 3.82, − 0.13)**
Perceived severity			
Mild	23.4 (5.8)	Ref	Ref
Moderate	22.2 (6.1)	−1.25 (−2.90, 0.38)	−0.66 (− 2.31, 0.99)
Severe	22.5 (6.1)	--0.86 (−2.50,0.77)	0.08 (−1.62, 1.78)
WHODAS Score		**−0.32 (− 0.48, − 0.17)**	**−0.36 (− 0.53, − 0.20)**

bold type signifies statistical significance at *p* < 0.05

## Discussion

In this community-based study from a rural, low-income country setting, women with PPD were found to be most likely to use religious and emotion-based coping strategies. Higher scores on emotion- and problem-based coping were associated with higher educational level, socio-economic status, urban residence and physical attribution to the symptoms. Dysfunctional coping was associated with marital problems. Emotion-based functioning was also increased in women who perceived their husbands to drink too much alcohol.

Religious coping has been found to be among the most frequently used coping strategy by people with depression and other stressors [47, 63–65]. In Ethiopia, most people affiliate themselves with a religion and, thus, it is consistent that religion emerged as the main resource for coping with symptoms of distress and stressful experiences in this study. Emotional coping, of which religious coping is an example, is usually understood to be employed when a person perceives the problem to be beyond their control [22]. However, religious coping has been conceptualized as either a mediator or a buffer for depressive symptoms, depending on how it is used by the individual [64–66]. An example of positive religious coping could be looking to God for strength, support or guidance. Negative religious coping on the other hand might involve feeling abandoned or being punished by God [67]. Negative religious coping has been found to be a strong predictor of worsening symptoms of depression [68]. In studies from Poland and Greece, women with symptoms of PPD were found to more often use emotion-focused coping [30, 33].

The least frequently used coping strategies by women with symptoms of PPD in this study were substance use or humor. This is in keeping with the high endorsement of religious coping strategies [69]. Moreover, the participants of this study are women who are less likely to be frequent alcohol users for socio-cultural reasons. The low levels of humor-related coping strategies- may reflect cultural norms, but also that this sample of women had high levels of depressive symptoms.

### The brief COPE scale and its construct validity

Confirmatory factor analysis supported the three dimensions of coping, with high loading values for most of the items except the substance use and venting items. The model fit was also fairly good, but fell short of the conventional cut-offs on the indices of model fit. It is possible that some aspects of coping which are relevant in this context were not captured fully by the scale. The Brief COPE scale has been validated in different cultures among women with breast cancer in Malaysia [39] and caregivers of people with dementia in the UK [40]. In both of these studies, the Brief COPE was reported to be valid and reliable. In the first validation study, sensitivity of the scale to change was also indicated, with the Effect Size Index (ESI) ranging from 0 to 0.53. In the second study, high internal consistency and test-retest reliability over a year were demonstrated for emotion-focused, problem-focused, and dysfunctional subscales.

### Factors associated with coping styles

There were very high correlations among the sub-scales of the COPE in this study, for example 0.98 between problem-focused and emotion-focused coping. The scores on all of the COPE sub-scales, problem-focused, emotion-focused and dysfunctional, were significantly higher for urban residents. Women with formal education were more likely to use both problem- and emotion-focused coping. Urban women may differ from rural women in characteristics such as their access to resources and information, and the way in which they express their feelings, all of which will influence coping styles. Women living in rural areas in Ethiopia are often less empowered than their urban counterparts, for example, more likely to live in a non-consenting marriage [70]. They are likely to have fewer resources for problem-focused coping, less propensity to express their private feelings for emotion-focused coping, and may have less personal freedom for dysfunctional coping. Women who perceived that their wealth was better than their neighbors were more likely to use emotion-focused, but not problem-focused, coping. In this rural Ethiopian context, social support is closely linked to a person’s capacity to reciprocate; therefore, poverty may undermine the possibility of obtaining emotional support and reframing of women’s difficulties [71].

Attribution of the symptoms of postpartum depression to a physical cause was associated with significantly higher scores for both problem and emotion-focused coping. However, being unable to identify a cause for the PPD symptoms was negatively associated with all three dimensions of coping. It follows that if a person does not know what caused their problem, this is likely to be a barrier to effective coping. One possible explanation is that women may not consider their PPD symptoms to be the result of an illness (and, therefore, to be a ‘problem’), but rather a part of life experience that they have to accept and live with. Women in LMICs attribute PPD to financial difficulties and marital conflict [72]. Similarly, in Ethiopia, although symptoms of PPD are recognized, they are attributed to social adversity or to failure of carrying out cultural chores that could expose to supernatural attacks which they believe cause the symptoms [73]. The implication of this may be that resources such as knowledge about depression and increasing social support might promote more problem-focused coping and contribute to improving symptoms.

Dysfunctional coping was increased in women who said that their relationship with their husbands was poor. Dysfunctional coping has been reported to be commonly used by people with high emotional distress, for example in the context of marital discord [74, 75]. The perception that their husbands drink too much alcohol was positively associated with emotion-focused coping. Thinking that one’s husband drinks too much alcohol may mean it is difficult to modify that behavior. This may lead to the perception of the problem as unsolvable. Those individuals who perceive that their stressor is uncontrollable tend to use either emotion-focused or dysfunctional coping strategies [22]. Women in Ethiopia especially in the rural parts get married at early age without their consent and are economically dependent on their husbands. They are expected to stoically tolerate marital problems and maintain their marriage at all costs. Women who are married at young age are unlikely to have been to school and less likely to have known about the marriage beforehand and more likely to have experienced forced first marital sex and subsequent intimate partner violence [70].

Women who reported more functional impairment (scoring higher on the WHODAS) were less likely to use emotion-focused and dysfunctional coping compared to those with less functional impairment. Furthermore, no association was seen between functional impairment and problem-focused coping. This finding was unexpected as more apparent disability would be expected to motivate women to engage in more overt coping actions. The WHODAS is a measure of disability linked to any health condition, not specific to mental health problems. However, we have shown previously in this context that postpartum depressive symptoms are independent of physical health status indicators, such as low hemoglobin or low body mass index [76], and are strongly associated with functional impairment [58]. It is possible that aspects of problem-solving coping in this context were not captured adequately with the COPE scale; however, further studies are required to better understand this association.

### Limitations

A limitation of this study is that we had not undertaken a formal cultural validation of the brief COPE for the local context. Nonetheless, the COPE was found to have reasonable construct validity in this sample. A further limitation is that it was not possible to elucidate the cause-effect relationship between depression and the coping strategies due to the cross-sectional nature of the study. A prospective study could help to elucidate whether coping strategies predispose women to depression or are a consequence of depression. The prognostic significance of particular coping strategies could also be investigated with a prospective design and would be an important evaluation of the predictive validity of the Brief COPE scale in this setting.

## Conclusion

Religious coping was the most commonly used coping strategy by women with symptoms of postpartum depression in this rural Ethiopian community. Although it is not possible to know whether coping strategies lead to depression or vice versa, interventions can be developed that build on existing coping strategies and enhance the capacity of women to use more adaptive coping. The Brief COPE scale has fair internal consistency and acceptable construct validity but further cultural validation is needed.

## Additional file

Additional file 1: Construct validity of brief COPE using confirmatory factor analysis. (DOCX 171 kb)

## Abbreviations

AMOS: Analysis of Moment Structure
CFI: Comparative Fit Index
COPE: Coping Orientation for Problems Experienced
IRB: Institutional Review Board
LMIC: Low and Middle-Income Country
OSSS: Oslo Social Support Scale
PHC: Primary Health Care
PHQ: Patient Health Questionnaire
PPD: Postpartum Depression
PRIME: Program for Improving Mental Health care
RMSEA: Root Mean Square Error of Approximation
SEMI: Short Explanatory Model Interview,
SNNPR: South Nations Nationalities and Peoples Region
TLI: Tucker Lewis Index
WHODAS: World Health Organization Disability Assessment Schedule
