# Supplementary material for: Coping strategies of women with postpartum depression symptoms in rural Ethiopia: a cross-sectional community study
Source: BMC Psychiatry. 2018 Feb 8;18:41. doi: 10.1186/s12888-018-1624-z (PMC5806287; doi:10.1186/s12888-018-1624-z)
Supplement: Supplementary file 1 — Construct validity of brief COPE using confirmatory factor analysis. (DOCX 171 kb) [file 12888_2018_1624_MOESM1_ESM.docx]

Brief COPE Scale

1 = I haven't been doing this at all 
 2 = I've been doing this a little bit 
 3 = I've been doing this a medium amount 
 4 = I've been doing this a lot

| № | Coping strategies | scores | | | |
| --- | --- | --- | --- | --- | --- |
|  |  | 1 | 2 | 3 | 4 |
| 1. | I've been turning to work or other activities to take my mind off things. |  |  |  |  |
| 2. | I've been concentrating my efforts on doing something about the situation I'm in. |  |  |  |  |
| 3. | I've been saying to myself "this isn't real.". |  |  |  |  |
| 4. | I've been using alcohol or other drugs to make myself feel better. |  |  |  |  |
| 5. | I've been getting emotional support from others. |  |  |  |  |
| 6. | I've been giving up trying to deal with it. |  |  |  |  |
| 7. | I've been taking action to try to make the situation better. |  |  |  |  |
| 8. | I've been refusing to believe that it has happened. |  |  |  |  |
| 9. | I've been saying things to let my unpleasant feelings escape. |  |  |  |  |
| 10. | I’ve been getting help and advice from other people. |  |  |  |  |
| 11. | I've been using alcohol or other drugs to help me get through it. |  |  |  |  |
| 12 | .  I've been trying to see it in a different light, to make it seem more positive. |  |  |  |  |
| 13. | I’ve been criticizing myself. |  |  |  |  |
| 14. | I've been trying to come up with a strategy about what to do. |  |  |  |  |
| 15 | I've been getting comfort and understanding from someone. |  |  |  |  |
| 16 | I've been giving up the attempt to cope. |  |  |  |  |
| 17. | I've been looking for something good in what is happening. |  |  |  |  |
| 18. | I've been making jokes about it. |  |  |  |  |
| 19. | I've been doing something to think about it less, such as going to movies, watching TV, reading, daydreaming, sleeping, or shopping. |  |  |  |  |
| 20. | I've been accepting the reality of the fact that it has happened. |  |  |  |  |
| 21 | I've been expressing my negative feelings. |  |  |  |  |
| 22 | I've been trying to find comfort in my religion or spiritual beliefs. |  |  |  |  |
| 23. | I’ve been trying to get advice or help from other people about what to do. |  |  |  |  |
| 24. | I've been learning to live with it. |  |  |  |  |
| 25. | I've been thinking hard about what steps to take. |  |  |  |  |
| 26. | I’ve been blaming myself for things that happened. |  |  |  |  |
| 27. | I've been praying or meditating. |  |  |  |  |
| 28. | I've been making fun of the situation. |  |  |  |  |

Scales are computed as follows (with no reversals of coding)

Self-distraction, items 1 and 19  **distraction, 2**
 Active coping, items 2 and 7  **activecope , 2**
Denial, items 3 and 8  **denial , 2**
Substance use, items 4 and 11  **subuse , 2**
Use of emotional support, items 5 and 15  **esupport 2**
Use of instrumental support, items 10 and 23  **isupport 2**
 Behavioral disengagement, items 6 and 16  **bdisengagement 2**
Venting, items 9 and 21  **venting 2**
 Positive reframing, items 12 and 17  **posreframing 2**
Planning, items 14 and 25  **planning**
Humor, items 18 and 28   **Humor**
Acceptance, items 20 and 24  **acceptance**
Religion, items 22 and 27  **religious**
Self-blame, items 13 and 26 Selfblame

The primary dimensions of the scale are classified by many into three as:

1. **Emotion focused coping**

- Emotional support(items 5 and 15) ….. **esupport 2**
- Religion( items 22 and 27) ………….. **religious**
- Positive reframing(items 22 and 27)(12, 17) **posreframing 2**
- Humor(items 18 and 28) …….. **Humor**
- Acceptance(items 20 and 24) … **acceptance**

1. **Problem focused coping**

- Active coping(items 2 and 7) … **activecope , 2**
- Planning(items 14 and 25 ) ….. **planning**
- Instrumental support(items 10 and 23) … **isupport 2**

1. **Dysfunctional/avoidance coping**

- Self-distraction(items 1 and 19 )… **distraction, 2**
- Denial(3 and 8 ) ………………… **denial , 2**
- Venting(items 9 and 21 ) ….. **venting 2**
- Substance use(items 4 and 11 ) …. **subuse , 2**
- Behavioral disengagement(items 6 and 16 ) .. **bdisengagement 2**
- Self-blame(13 and 26)  ……. **Selfblame**


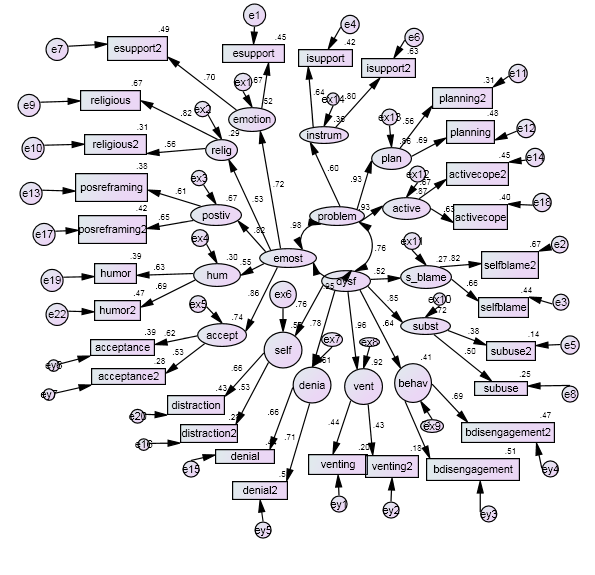


**Result (Default model)**

Minimum was achieved

Chi-square = 1192.237

Degrees of freedom = 333

Probability level = .000

**Model Fit Summary**

**CMIN**

| Model | NPAR | CMIN | DF | P | CMIN/DF |
| --- | --- | --- | --- | --- | --- |
| Default model | 101 | 1192.237 | 333 | .000 | 3.580 |
| Saturated model | 434 | .000 | 0 |  |  |
| Independence model | 28 | 3437.558 | 406 | .000 | 8.467 |

**Baseline Comparisons**

| Model | NFI Delta1 | RFI rho1 | IFI Delta2 | TLI rho2 | CFI |
| --- | --- | --- | --- | --- | --- |
| Default model | .653 | .577 | .723 | **.654** | **.717** |
| Saturated model | 1.000 |  | 1.000 |  | 1.000 |
| Independence model | .000 | .000 | .000 | .000 | .000 |

**Parsimony-Adjusted Measures**

| Model | PRATIO | PNFI | PCFI |
| --- | --- | --- | --- |
| Default model | .820 | .536 | .588 |
| Saturated model | .000 | .000 | .000 |
| Independence model | 1.000 | .000 | .000 |

**NCP**

| Model | NCP | LO 90 | HI 90 |
| --- | --- | --- | --- |
| Default model | 859.237 | 757.623 | 968.415 |
| Saturated model | .000 | .000 | .000 |
| Independence model | 3031.558 | 2848.072 | 3222.401 |

**FMIN**

| Model | FMIN | F0 | LO 90 | HI 90 |
| --- | --- | --- | --- | --- |
| Default model | .379 | .273 | .241 | .308 |
| Saturated model | .000 | .000 | .000 | .000 |
| Independence model | 1.093 | .964 | .905 | 1.024 |

**RMSEA**

| Model | RMSEA | LO 90 | HI 90 | PCLOSE |
| --- | --- | --- | --- | --- |
| Default model | **.029** | **.027** | **.030** | 1.000 |
| Independence model | .049 | .047 | .050 | .918 |

**AIC**

| Model | AIC | BCC | BIC | CAIC |
| --- | --- | --- | --- | --- |
| Default model | 1394.237 | 1396.116 |  |  |
| Saturated model | 868.000 | 876.076 |  |  |
| Independence model | 3493.558 | 3494.079 |  |  |

**ECVI**

| Model | ECVI | LO 90 | HI 90 | MECVI |
| --- | --- | --- | --- | --- |
| Default model | .443 | .411 | .478 | .444 |
| Saturated model | .276 | .276 | .276 | .278 |
| Independence model | 1.110 | 1.052 | 1.171 | 1.111 |

**HOELTER**

| Model | HOELTER .05 | HOELTER .01 |
| --- | --- | --- |
| Default model | 994 | 1045 |
| Independence model | 416 | 435 |

**Estimates (Group number 1 - Default model)**

**Scalar Estimates (Group number 1 - Default model)**

**Maximum Likelihood Estimates**

**Regression Weights: (Group number 1 - Default model)**

|  |  |  | Estimate | S.E. | C.R. | P | Label |
| --- | --- | --- | --- | --- | --- | --- | --- |
| active | <--- | problem | 1.243 | .165 | 7.544 | *** |  |
| plan | <--- | problem | 1.251 | .158 | 7.924 | *** |  |
| instrum | <--- | problem | 1.000 |  |  |  |  |
| accept | <--- | emost | 1.006 | .131 | 7.662 | *** |  |
| hum | <--- | emost | .428 | .076 | 5.641 | *** |  |
| postiv | <--- | emost | .946 | .128 | 7.373 | *** |  |
| relig | <--- | emost | .866 | .129 | 6.731 | *** |  |
| emotion | <--- | emost | 1.000 |  |  |  |  |
| self | <--- | dysf | 1.000 |  |  |  |  |
| denia | <--- | dysf | 1.013 | .142 | 7.143 | *** |  |
| vent | <--- | dysf | .830 | .132 | 6.298 | *** |  |
| behav | <--- | dysf | .773 | .117 | 6.595 | *** |  |
| subst | <--- | dysf | .464 | .074 | 6.262 | *** |  |
| s_blame | <--- | dysf | .652 | .122 | 5.324 | *** |  |
| esupport2 | <--- | emotion | .981 | .111 | 8.828 | *** |  |
| esupport | <--- | emotion | 1.000 |  |  |  |  |
| religious | <--- | relig | 1.000 |  |  |  |  |
| religious2 | <--- | relig | .609 | .102 | 5.980 | *** |  |
| posreframing | <--- | postiv | 1.000 |  |  |  |  |
| posreframing2 | <--- | postiv | 1.152 | .133 | 8.667 | *** |  |
| humor | <--- | hum | 1.000 |  |  |  |  |
| humor2 | <--- | hum | 1.105 | .178 | 6.224 | *** |  |
| acceptance | <--- | accept | 1.000 |  |  |  |  |
| acceptance2 | <--- | accept | .848 | .108 | 7.838 | *** |  |
| planning2 | <--- | plan | .849 | .096 | 8.829 | *** |  |
| planning | <--- | plan | 1.000 |  |  |  |  |
| activecope2 | <--- | active | 1.076 | .111 | 9.717 | *** |  |
| distraction | <--- | self | 1.000 |  |  |  |  |
| distraction2 | <--- | self | .840 | .126 | 6.681 | *** |  |
| denial | <--- | denia | 1.000 |  |  |  |  |
| denial2 | <--- | denia | 1.046 | .116 | 9.020 | *** |  |
| venting | <--- | vent | 1.000 |  |  |  |  |
| venting2 | <--- | vent | .975 | .170 | 5.725 | *** |  |
| bdisengagement | <--- | behav | 1.000 |  |  |  |  |
| bdisengagement2 | <--- | behav | .920 | .120 | 7.690 | *** |  |
| selfblame | <--- | s_blame | 1.000 |  |  |  |  |
| selfblame2 | <--- | s_blame | 1.224 | .181 | 6.762 | *** |  |
| isupport2 | <--- | instrum | 1.000 |  |  |  |  |
| activecope | <--- | active | 1.000 |  |  |  |  |
| subuse | <--- | subst | 1.000 |  |  |  |  |
| subuse2 | <--- | subst | .659 | .133 | 4.944 | *** |  |
| isupport | <--- | instrum | .779 | .103 | 7.543 | *** |  |

**Standardized Regression Weights: (Group number 1 - Default model)**

|  |  |  | Estimate |
| --- | --- | --- | --- |
| active | <--- | problem | .930 |
| plan | <--- | problem | .928 |
| instrum | <--- | problem | .603 |
| accept | <--- | emost | .860 |
| hum | <--- | emost | .549 |
| postiv | <--- | emost | .821 |
| relig | <--- | emost | .535 |
| emotion | <--- | emost | .721 |
| self | <--- | dysf | .757 |
| denia | <--- | dysf | .779 |
| vent | <--- | dysf | .960 |
| behav | <--- | dysf | .638 |
| subst | <--- | dysf | .846 |
| s_blame | <--- | dysf | .516 |
| esupport2 | <--- | emotion | .699 |
| esupport | <--- | emotion | .671 |
| religious | <--- | relig | .818 |
| religious2 | <--- | relig | .556 |
| posreframing | <--- | postiv | .613 |
| posreframing2 | <--- | postiv | .647 |
| humor | <--- | hum | .627 |
| humor2 | <--- | hum | .687 |
| acceptance | <--- | accept | .623 |
| acceptance2 | <--- | accept | .533 |
| planning2 | <--- | plan | .556 |
| planning | <--- | plan | .694 |
| activecope2 | <--- | active | .669 |
| distraction | <--- | self | .659 |
| distraction2 | <--- | self | .528 |
| denial | <--- | denia | .663 |
| denial2 | <--- | denia | .714 |
| venting | <--- | vent | .444 |
| venting2 | <--- | vent | .427 |
| bdisengagement | <--- | behav | .713 |
| bdisengagement2 | <--- | behav | .686 |
| selfblame | <--- | s_blame | .660 |
| selfblame2 | <--- | s_blame | .820 |
| isupport2 | <--- | instrum | .796 |
| activecope | <--- | active | .632 |
| subuse | <--- | subst | .499 |
| subuse2 | <--- | subst | .380 |
| isupport | <--- | instrum | .645 |

**Intercepts: (Group number 1 - Default model)**

|  |  |  | Estimate | S.E. | C.R. | P | Label |
| --- | --- | --- | --- | --- | --- | --- | --- |
| esupport |  |  | 2.047 | .057 | 35.623 | *** |  |
| esupport2 |  |  | 2.683 | .054 | 49.615 | *** |  |
| religious |  |  | 2.816 | .055 | 51.211 | *** |  |
| religious2 |  |  | 3.177 | .049 | 64.448 | *** |  |
| posreframing |  |  | 2.127 | .052 | 40.702 | *** |  |
| posreframing2 |  |  | 1.995 | .057 | 34.976 | *** |  |
| humor |  |  | 1.213 | .035 | 35.142 | *** |  |
| humor2 |  |  | 1.210 | .035 | 34.732 | *** |  |
| isupport |  |  | 2.101 | .051 | 41.546 | *** |  |
| isupport2 |  |  | 2.481 | .053 | 47.151 | *** |  |
| planning2 |  |  | 2.481 | .052 | 47.736 | *** |  |
| planning |  |  | 2.483 | .049 | 50.650 | *** |  |
| activecope2 |  |  | 2.600 | .054 | 47.910 | *** |  |
| activecope |  |  | 2.418 | .053 | 45.331 | *** |  |
| distraction |  |  | 2.475 | .056 | 43.858 | *** |  |
| distraction2 |  |  | 2.460 | .059 | 41.617 | *** |  |
| denial |  |  | 1.844 | .055 | 33.440 | *** |  |
| selfblame2 |  |  | 2.278 | .053 | 42.927 | *** |  |
| selfblame |  |  | 2.203 | .054 | 40.878 | *** |  |
| subuse2 |  |  | 1.125 | .027 | 42.047 | *** |  |
| subuse |  |  | 1.161 | .031 | 37.503 | *** |  |
| acceptance |  |  | 2.208 | .052 | 42.315 | *** |  |
| acceptance2 |  |  | 1.930 | .052 | 37.373 | *** |  |
| denial2 |  |  | 1.790 | .054 | 33.378 | *** |  |
| bdisengagement |  |  | 1.745 | .048 | 36.489 | *** |  |
| bdisengagement2 |  |  | 1.584 | .046 | 34.668 | *** |  |
| venting |  |  | 2.203 | .055 | 40.229 | *** |  |
| venting2 |  |  | 1.751 | .055 | 31.556 | *** |  |

**Covariances: (Group number 1 - Default model)**

|  |  |  | Estimate | S.E. | C.R. | P | Label |
| --- | --- | --- | --- | --- | --- | --- | --- |
| problem | <--> | emost | .265 | .043 | 6.180 | *** |  |
| dysf | <--> | emost | .285 | .046 | 6.241 | *** |  |
| dysf | <--> | problem | .207 | .036 | 5.756 | *** |  |

**Correlations: (Group number 1 - Default model)**

|  |  |  | Estimate |
| --- | --- | --- | --- |
| problem | <--> | emost | .981 |
| dysf | <--> | emost | .946 |
| dysf | <--> | problem | .756 |

**Variances: (Group number 1 - Default model)**

|  |  |  | Estimate | S.E. | C.R. | P | Label |
| --- | --- | --- | --- | --- | --- | --- | --- |
| problem |  |  | .245 | .054 | 4.525 | *** |  |
| emost |  |  | .297 | .063 | 4.735 | *** |  |
| dysf |  |  | .305 | .065 | 4.677 | *** |  |
| ex1 |  |  | .274 | .061 | 4.525 | *** |  |
| ex6 |  |  | .227 | .078 | 2.923 | .003 |  |
| ex7 |  |  | .202 | .051 | 3.932 | *** |  |
| ex8 |  |  | .018 | .054 | .331 | .741 |  |
| ex10 |  |  | .026 | .023 | 1.134 | .257 |  |
| ex11 |  |  | .357 | .068 | 5.229 | *** |  |
| ex12 |  |  | .059 | .041 | 1.421 | .155 |  |
| ex13 |  |  | .062 | .047 | 1.312 | .189 |  |
| ex14 |  |  | .429 | .086 | 4.973 | *** |  |
| ex9 |  |  | .265 | .055 | 4.792 | *** |  |
| ex4 |  |  | .126 | .028 | 4.428 | *** |  |
| ex5 |  |  | .106 | .052 | 2.025 | .043 |  |
| ex3 |  |  | .129 | .043 | 2.988 | .003 |  |
| ex2 |  |  | .556 | .126 | 4.406 | *** |  |
| e1 |  |  | .699 | .077 | 9.110 | *** |  |
| e20 |  |  | .694 | .092 | 7.549 | *** |  |
| e16 |  |  | .970 | .089 | 10.947 | *** |  |
| e3 |  |  | .631 | .081 | 7.803 | *** |  |
| e2 |  |  | .355 | .103 | 3.429 | *** |  |
| e18 |  |  | .657 | .062 | 10.613 | *** |  |
| e14 |  |  | .626 | .065 | 9.697 | *** |  |
| e11 |  |  | .718 | .061 | 11.711 | *** |  |
| e7 |  |  | .576 | .069 | 8.297 | *** |  |
| e13 |  |  | .656 | .063 | 10.357 | *** |  |
| e10 |  |  | .646 | .064 | 10.034 | *** |  |
| e9 |  |  | .385 | .123 | 3.125 | .002 |  |
| e17 |  |  | .728 | .077 | 9.488 | *** |  |
| e19 |  |  | .278 | .034 | 8.157 | *** |  |
| e22 |  |  | .247 | .038 | 6.483 | *** |  |
| e4 |  |  | .575 | .064 | 8.923 | *** |  |
| ey5 |  |  | .543 | .068 | 8.005 | *** |  |
| e15 |  |  | .656 | .069 | 9.465 | *** |  |
| ey1 |  |  | .926 | .086 | 10.813 | *** |  |
| ey2 |  |  | .968 | .086 | 11.197 | *** |  |
| ey3 |  |  | .434 | .062 | 7.001 | *** |  |
| ey4 |  |  | .426 | .055 | 7.793 | *** |  |
| e5 |  |  | .236 | .020 | 11.929 | *** |  |
| e6 |  |  | .391 | .086 | 4.545 | *** |  |
| e12 |  |  | .480 | .057 | 8.381 | *** |  |
| e8 |  |  | .277 | .031 | 9.042 | *** |  |
| ey6 |  |  | .641 | .069 | 9.286 | *** |  |
| ey7 |  |  | .734 | .065 | 11.380 | *** |  |

**Squared Multiple Correlations: (Group number 1 - Default model)**

|  |  |  | Estimate |
| --- | --- | --- | --- |
| subst |  |  | .715 |
| s_blame |  |  | .266 |
| behav |  |  | .407 |
| vent |  |  | .922 |
| denia |  |  | .607 |
| self |  |  | .573 |
| instrum |  |  | .364 |
| active |  |  | .866 |
| plan |  |  | .861 |
| accept |  |  | .740 |
| hum |  |  | .302 |
| postiv |  |  | .674 |
| relig |  |  | .286 |
| emotion |  |  | .520 |
| subuse |  |  | .249 |
| isupport2 |  |  | .633 |
| subuse2 |  |  | .145 |
| bdisengagement2 |  |  | .470 |
| bdisengagement |  |  | .508 |
| venting2 |  |  | .183 |
| venting |  |  | .197 |
| denial |  |  | .440 |
| denial2 |  |  | .509 |
| planning |  |  | .482 |
| isupport |  |  | .416 |
| acceptance2 |  |  | .285 |
| acceptance |  |  | .388 |
| humor2 |  |  | .471 |
| humor |  |  | .393 |
| posreframing2 |  |  | .419 |
| religious |  |  | .669 |
| religious2 |  |  | .309 |
| posreframing |  |  | .376 |
| esupport2 |  |  | .488 |
| planning2 |  |  | .309 |
| activecope2 |  |  | .447 |
| activecope |  |  | .400 |
| selfblame2 |  |  | .673 |
| selfblame |  |  | .435 |
| distraction2 |  |  | .279 |
| distraction |  |  | .434 |
| esupport |  |  | .450 |
